# Radiomics analysis of ultrasound images to discriminate between benign and malignant adnexal masses with solid morphology on ultrasound

**DOI:** 10.1002/uog.27680

**Published:** 2025-02-02

**Authors:** F. Moro, M. Vagni, H. E. Tran, F. Bernardini, F. Mascilini, F. Ciccarone, C. Nero, D. Giannarelli, L. Boldrini, A. Fagotti, G. Scambia, L. Valentin, A. C. Testa

**Affiliations:** ^1^ Dipartimento Scienze della Salute della Donna, del Bambino e di Sanità Pubblica Fondazione Policlinico Universitario A. Gemelli IRCCS Rome Italy; ^2^ Istituto di Radiologia Università Cattolica del Sacro Cuore Rome Italy; ^3^ Dipartimento di Diagnostica per Immagini, Radioterapia Oncologica ed Ematologia Fondazione Policlinico Universitario A. Gemelli IRCCS Rome Italy; ^4^ Epidemiology and Biostatistics Facility, G‐STeP Generator Fondazione Policlinico Universitario A. Gemelli IRCCS Rome Italy; ^5^ Dipartimento Universitario Scienze della Vita e Sanità Pubblica Università Cattolica del Sacro Cuore Rome Italy; ^6^ Department of Obstetrics and Gynecology Skåne University Hospital Malmö Sweden; ^7^ Department of Clinical Sciences Lund University Malmö Sweden

**Keywords:** artificial intelligence, machine learning, ovarian tumors, ultrasonography

## Abstract

**Objective:**

The primary aim was to identify radiomics ultrasound features that can distinguish between benign and malignant adnexal masses with solid ultrasound morphology, and between primary malignant (including borderline and primary invasive) and metastatic solid ovarian masses, and to develop ultrasound‐based machine learning models that include radiomics features to discriminate between benign and malignant solid adnexal masses. The secondary aim was to compare the discrimination performance of our newly developed radiomics models with that of the Assessment of Different NEoplasias in the adneXa (ADNEX) model and that of subjective assessment by an experienced ultrasound examiner.

**Methods:**

This was a retrospective, observational single‐center study conducted at Fondazione Policlinico Universitario A. Gemelli IRCC, in Rome, Italy. Included were patients with a histological diagnosis of an adnexal tumor with solid morphology according to International Ovarian Tumor Analysis (IOTA) terminology at preoperative ultrasound examination performed in 2014–2020, who were managed with surgery. The patient cohort was split randomly into training and validation sets at a ratio of 70:30 and with the same proportion of benign and malignant tumors in the two subsets, with malignant tumors including borderline, primary invasive and metastatic tumors. We extracted 68 radiomics features, belonging to two different families: intensity‐based statistical features and textural features. Models to predict malignancy were built based on a random forest classifier, fine‐tuned using 5‐fold cross‐validation over the training set, and tested on the held‐out validation set. The variables used in model‐building were patient age and radiomics features that were statistically significantly different between benign and malignant adnexal masses and assessed as not redundant based on the Pearson correlation coefficient. We evaluated the discriminative ability of the models and compared it to that of the ADNEX model and that of subjective assessment by an experienced ultrasound examiner using the area under the receiver‐operating‐characteristics curve (AUC) and classification performance by calculating sensitivity and specificity.

**Results:**

In total, 326 patients were included and 775 preoperative ultrasound images were analyzed. Of the 68 radiomics features extracted, 52 differed statistically significantly between benign and malignant tumors in the training set, and 18 uncorrelated features were selected for inclusion in model‐building. The same 52 radiomics features differed significantly between benign, primary malignant and metastatic tumors. However, the values of the features manifested overlapped between primary malignant and metastatic tumors and did not differ significantly between them. In the validation set, 25/98 (25.5%) tumors were benign and 73/98 (74.5%) were malignant (6 borderline, 57 primary invasive, 10 metastatic). In the validation set, a model including only radiomics features had an AUC of 0.80, sensitivity of 0.78 and specificity of 0.76 at an optimal cut‐off for risk of malignancy of 68%, based on Youden's index. The corresponding results for a model including age and radiomics features were AUC of 0.79, sensitivity of 0.86 and specificity of 0.56 (cut‐off 60%, based on Youden's index), while those of the ADNEX model were AUC of 0.88, sensitivity of 0.99 and specificity of 0.64 (at a 20% risk‐of‐malignancy cut‐off). Subjective assessment had a sensitivity of 0.99 and specificity of 0.72.

**Conclusions:**

Our radiomics model had moderate discriminative ability on internal validation and the addition of age to this model did not improve its performance. Even though our radiomics models had discriminative ability inferior to that of the ADNEX model, our results are sufficiently promising to justify continued development of radiomics analysis of ultrasound images of adnexal masses. © 2024 The Author(s). *Ultrasound in Obstetrics & Gynecology* published by John Wiley & Sons Ltd on behalf of International Society of Ultrasound in Obstetrics and Gynecology.

## INTRODUCTION

It has been reported that 13% of ovarian masses detected on ultrasound in referral centers and subsequently removed by surgery are classified as solid on ultrasound according to the International Ovarian Tumor Analysis (IOTA) terminology, and that 62% of such solid tumors are malignant^1^. About 30% of solid malignant masses may be metastases from non‐ovarian tumors^2^. Ultrasound imaging provides useful information to characterize adnexal masses as benign or malignant, and there are several different ultrasound methods to classify them. One is subjective assessment of the ultrasound image (pattern recognition), which is an excellent method in the hands of an experienced examiner^3^. Others are mathematical models, such as the Assessment of Different NEoplasias in the adneXa (ADNEX) model^4^. A unique ability of the ADNEX model is that it calculates the likelihood of four different types of malignancy (borderline tumor, Stage‐I or Stage II−IV ovarian cancer, or a metastasis in the ovary from another primary tumor). On external validation, the ability of the ADNEX model to discriminate between benign and malignant masses has been shown to be very good and the model has been shown to be well‐calibrated (i.e. the calculated risk of malignancy agrees well with the observed prevalence of malignancy)^5^. The ADNEX model maintains its diagnostic performance in the hands of operators with different experience and training^6^.

Discrimination between primary ovarian malignancies and metastatic tumors in the ovary is clinically important for planning adequate management. Surgical cytoreduction is the treatment of choice for primary ovarian malignancies, while the impact of surgery on the clinical outcome of metastatic tumors in the ovaries is controversial^7^, ^8^, ^9^. Differences in sonographic findings between primary ovarian malignancies and metastases in the ovaries have been described^2^, ^10^. Most metastatic tumors arising from the breast, endometrium and stomach appear solid on ultrasound examination^10^. However, many primary invasive ovarian malignancies (e.g. high‐grade serous carcinoma, carcinosarcoma, malignant germ‐cell tumors, sex‐cord‐stromal tumors and steroid cell tumors)^11^, ^12^, ^13^, ^14^, ^15^ also manifest solid ultrasound morphology, as do many benign ovarian sex‐cord‐stromal tumors, such as fibromas and thecomas^16^. Therefore, it is challenging even for an experienced ultrasound examiner to classify solid ovarian tumors correctly using subjective assessment.

In recent years, there has been growing interest in the use of radiomics, a technique to extract, analyze and interpret quantitative data from medical images. The association between radiomics data and tumor diagnosis, prognosis and genetic information is being investigated^17^. The application of radiomics‐based predictive models for ultrasound faces several challenges, including variability in image acquisition protocols and ultrasound devices, operator dependency in segmenting the region of interest (ROI), noisy images and the need for large, annotated datasets to train robust machine‐learning models^18^. The choice of method for feature selection and model optimization and that of validation strategy affects generalizability and reliability of the developed models.

Several studies have evaluated the ability of radiomics analysis of magnetic resonance imaging^19^ and computed tomography^20^, ^21^ images to classify ovarian masses as benign or malignant or to predict therapeutic response and prognosis. Few data are available on the use of radiomics to extract information from ultrasound images of ovarian masses. We previously used radiomics analysis of ultrasound images to develop a radiogenomic model for predicting germline *BRCA1/2* gene status in women with apparently normal ovaries on ultrasound^22^. Chiappa *et al*.^23^ applied radiomics analysis to ultrasound images of ovarian masses to develop a model to predict malignancy in an adnexal mass. Christiansen *et al*.^24^ demonstrated the efficacy of deep neural networks in distinguishing benign from malignant ovarian tumors, achieving a level of accuracy comparable to that of subjective assessment by an expert. They used a rectangular ROI so that manual segmentation of the tumor was not needed^24^.

The primary aim of this study was to identify radiomics ultrasound features that can distinguish between benign and malignant adnexal masses with solid ultrasound morphology, and between primary malignant (including borderline and primary invasive) and metastatic solid ovarian masses, and to develop ultrasound‐based machine‐learning models that include radiomics features to discriminate between benign and malignant solid adnexal masses. The secondary aim was to compare the discrimination performance of the newly developed radiomics models with that of the ADNEX model and that of subjective assessment by an experienced ultrasound examiner.

## METHODS

This was a retrospective observational, single‐center study conducted at Fondazione Policlinico Universitario A. Gemelli IRCC, in Rome, Italy. Patients with an adnexal tumor with solid morphology according to IOTA terminology^25^ on ultrasound examination performed between 2014 and 2021 were identified by searching the IOTA database (IOTA phase 5^26^ and IOTA phase 7, both being ongoing prospective observational studies, and the latter registered at ClinicalTrials.gov NCT02847832). The IOTA database is a research database that includes all patients who participated in the international multicenter IOTA studies. It contains clinical, ultrasound and histological data of patients with an adnexal mass examined with transvaginal ultrasound (supplemented with abdominal ultrasound if needed) before surgery and follow‐up data for non‐surgically managed patients. The IOTA phase‐7 study was designed to create and validate risk prediction models to discriminate between benign and malignant adnexal tumors, while the primary aim of the IOTA phase‐5 study was to elucidate the natural history of adnexal masses with benign ultrasound morphology managed conservatively^26^. In this study, we included only patients managed with surgery. Written informed consent was obtained from each patient.

The ultrasound examinations were carried out using the standardized IOTA examination and measurement technique and the ultrasound images of the adnexal masses were described using the standardized IOTA terminology^25^. All ultrasound examiners were experienced (level II or III according to the European Federation of Societies for Ultrasound in Medicine and Biology (EFSUMB)^27^), and all ultrasound examinations were carried out using high‐end ultrasound equipment (Voluson E10 machine, GE Healthcare, Waukesha, WI, USA; HERA W10 and HERA I10 machine, Samsung Medison Co., Ltd., Gyeonggi‐do, Korea). The frequency of the transvaginal probes varied between 5.0 and 9.0 MHz and that of the transabdominal probes between 3.5 and 5.0 MHz.

Information on the ultrasound results, including those of subjective assessment (pattern recognition) by the original ultrasound examiner, and on the histological diagnosis and stage according to the International Federation of Gynecology and Obstetrics (FIGO)^28^ was obtained from the IOTA database. The histological diagnosis was subsequently verified in the patients' records.

Patient inclusion criteria were: age at least 18 years; solid adnexal mass according to IOTA terminology (i.e. at least 80% of the tumor estimated to consist of solid tissue on ultrasound^25^); at least one Digital Imaging and Communications in Medicine (DICOM) ultrasound image of the solid tumor; surgery performed within 120 days after the ultrasound examination. Patients were excluded if the only images available were printed images, or DICOM images in which the external margins of the ovarian tumor were not well visualized, or calipers or text covered the tumor.

### Image segmentation

**Figure 1:**
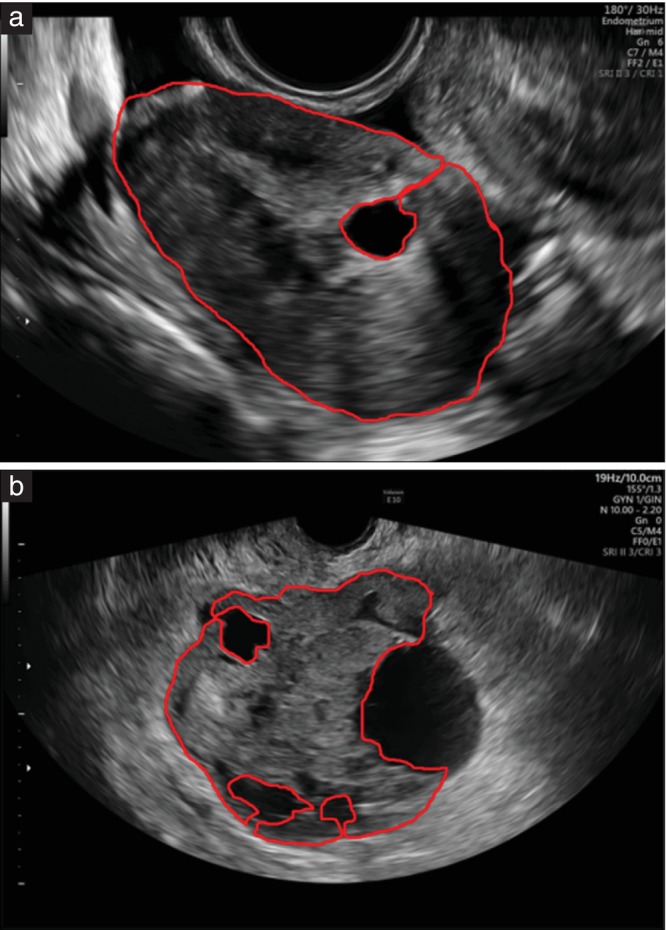
Ultrasound images with segmented tumor masks (red contour), in benign (a) and malignant (b) solid adnexal masses. Only solid tissue was included in the region of interest, and cystic areas larger than 5 mm in maximum diameter were excluded.

Transabdominal and transvaginal grayscale ultrasound images of the solid tumors saved in DICOM format were retrieved and used in our analysis. The images were screened for quality by an expert gynecologist (F.M.). The ROI of each image was segmented manually by a single trained gynecologist (F.B., EFSUMB level II^27^) using the software Aliza, version 1.48 (Aliza Medical Imaging, Bonn, Germany). The ROI included only the solid component of the mass. If the solid mass contained cystic areas, the examiner excluded from the ROI cysts that were larger than 5 mm in maximum diameter (Figure 1). This is because we wanted to analyze the ultrasound characteristics of the solid tissue itself.

### Extraction of radiomics features

**Figure 2:**
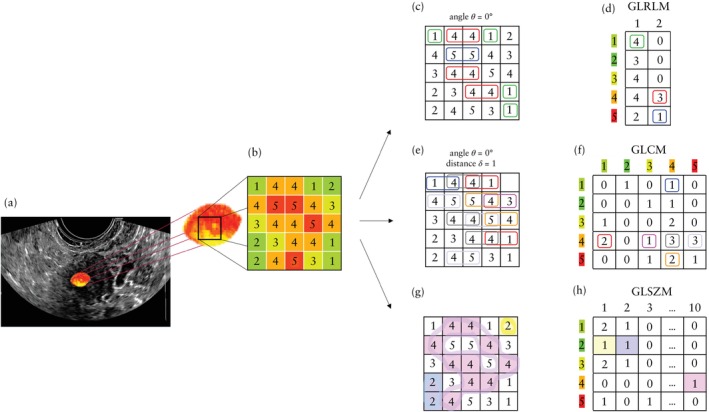
Example of an ultrasound image (a) with textural matrices constructed based on the spatial arrangement of gray levels (b). Matrices in (c), (e) and (g) are the same as matrix in (b), with details on how the textural matrices are then computed. The gray‐level run length matrix (GLRLM) (d) represents consecutive pixels with the same intensity along one direction. Rows indicate gray levels (1 to 5) and columns indicate the length of the path containing pixels with the same gray level. For each element of the matrix in (d), the row indicates the occurrence of gray level, and the column indicates the run length along a horizontal direction (defined by angle θ = 0° in matrix (c)). For example, the red box in matrix (d) indicates that there are three groups of dimension 2 with gray‐level 4 in the original gray‐level matrix (c). The gray‐level co‐occurrence matrix (GLCM) (f) assesses co‐occurrence of gray levels within neighboring pixels along one direction. Both rows and columns indicate gray levels. Each element of the matrix in (f) contains the frequency at which combinations of grey levels given by row and column occur in neighboring pixels along a horizontal direction from left to right. For example, the red box in matrix (f) indicates that there are two groups of consecutive pixels 4 and 1 in the original gray‐level matrix (e) along the horizontal right direction. The gray‐level size zone matrix (GLSZM) (h) counts the number of groups of connected pixels with the same gray level. Rows indicate gray levels and columns indicate the size of the zone containing neighboring pixels in any direction that have the same gray level. Each element of the matrix in (h) represents the number of zones with gray level given by the row, and size given by the column. For example, the pink box in matrix (h) indicates that there is one group composed of 10 neighboring pixels with gray‐level 4 in the original gray‐level matrix (g). Textural features are computed from the gray‐level matrices^31^.

Radiomics features were extracted for quantitative image analysis using MODDICOM^29^, an open‐source in‐house software solution developed by the Radiomics Research Core facility of Fondazione Policlinico Universitario A. Gemelli IRCCS, fully compliant with the Image Biomarker Standardization Initiative recommendations^30^. We extracted 68 radiomics features belonging to two different families: intensity‐based statistical features (F_stat) and textural features. The statistical features described the statistical properties of the gray‐level histogram within the ROI (e.g. mean, median, variance, skewness, kurtosis), while the textural features provided information on the spatial distribution of the gray levels within the ROI. The textural features were computed^31^ based on the following matrices: (1) gray‐level size zone matrix (F_szm or GLSZM), which represents the number of groups (zones) of neighboring pixels with the same gray level; (2) gray‐level run length matrix (F_rlm or GLRLM), which represents the length of a consecutive sequence of pixels with the same gray level; (3) gray‐level co‐occurrence matrix (F_cm or GLCM), in which the co‐occurrence of gray levels within neighboring pixels is reported^32^ (Figure 2).

### Statistical analysis and modeling

Statistical analysis and modeling were performed using RStudio^33^ (R version 3.6), an integrated development environment for R, and the Python programming language (version 3.9)^34^, a high‐level programming language widely used for data analysis and scientific computing.

Categorical variables are described as absolute frequencies and percentages, while continuous variables are summarized using either the median, interquartile range (25^th^ and 75^th^ percentiles) and range (minimum and maximum values), or the mean and SD. The statistical significance of differences between benign and malignant tumors was assessed using the chi‐square test for categorical variables (or Fisher's exact test when appropriate) and the Wilcoxon–Mann–Whitney test for continuous variables. The statistical significance of differences between benign, primary malignant and metastatic tumors was calculated using the chi‐square test for categorical variables (or Fisher's exact test when appropriate) and the Kruskal–Wallis test for continuous variables.

For the radiomics analysis, all ultrasound images from the same tumor were used to extract radiomics features. In case of multiple images of the same adnexal tumor, the mean value of each radiomics feature across the images was used as being representative of the tumor. In accordance with IOTA study protocols, if there were bilateral solid masses, the largest (dominant) mass was used for our primary objective. The Wilcoxon–Mann–Whitney test was used to identify radiomics features that differed statistically significantly between benign and malignant tumors in the training set (two‐tailed *P*‐value < 0.05 was considered statistically significant). We adjusted the *P*‐values for multiple testing using the Benjamini–Hochberg procedure, with the significance level set at 0.05.

#### Model development and internal validation

To develop a radiomics model to predict the risk of malignancy, the cohort of patients was split randomly into training and hold‐out validation sets with a ratio of 70:30, and with the same proportion of benign and malignant tumors in the two subsets (with borderline tumors classified as malignant). For a number of radiomics features of around 70 and an experimental‐wise error rate of 0.05, a training set with a sample size of 228 patients would have a power of 80% to detect a true difference in feature expression of at least 0.6, measured as Cohen's d effect size^35^.

The correlation between radiomics features that exhibited statistically significant differences between benign and malignant tumors (Wilcoxon–Mann–Whitney and Benjamini–Hochberg tests) was investigated using the Pearson correlation coefficient. If a feature was highly correlated with any other feature (correlation coefficient > 0.9), one of the two was selected randomly to be used in model‐building, while the other was not used. To bring the values of the selected radiomics features to a common scale, they were normalized to the range of [0,1] using min–max scaling. For building the radiomics models, we tested four classifiers: linear regressor, random forest, extreme gradient boosting (XGBoost) and support vector machine. Among them, the random‐forest classifier exhibited superior performance (Table S1). Therefore, we present our results based on a random‐forest classifier, which works by creating an ensemble of different decision trees^36^. To identify the optimal set of hyperparameters (parameters characteristic of the machine‐learning model, which modify and guide the learning process), fine‐tuning was performed with a randomized grid search using a 5‐fold cross‐validation over the training set.

Two models were developed to predict the risk of malignancy. The first model included only the selected radiomics features as input variables (radiomics‐only model). The second model included the selected radiomics features plus patient age (age + radiomics model). Age was added, because it is known to affect the risk of malignancy. Moreover, it is easy to retrieve information on age, and age is a predictor in the ADNEX model to which we compared the radiomics models.

The two radiomics models were tested on the hold‐out validation set. Their ability to discriminate between benign and malignant masses was described by the area under the receiver‐operating‐characteristics curve (AUC). We also calculated sensitivity, specificity, positive likelihood ratio and negative likelihood ratio when using the best cut‐off to classify the mass as malignant (the cut‐off providing the largest number of correct classifications according to Youden's index^37^) with their 95% CIs. We used normal approximation to estimate the 95% CIs for all metrics except AUC. To estimate the 95% CI of the AUC we used bootstrapping. The discriminative performance of the radiomics models in the validation set was compared between tumors with only one image and tumors with multiple images.

#### Comparison of discriminative performance

The diagnostic performance of the two radiomics models was compared with that of the ADNEX model and with subjective assessment by the original ultrasound examiner. The ADNEX model can be used with or without information on CA125 serum levels. We compared the radiomics models with the ADNEX model without CA125, which included the following eight predictors: age, type of center (oncological referral center or other), maximum diameter of the lesion (log transformed), proportion of solid tissue (calculated as the ratio between the largest diameter of the largest solid component and the largest diameter of the lesion, quadratic term), number of papillary projections, more than 10 cyst locules (yes or no), acoustic shadows (yes or no) and ascites (yes or no)^4^. We applied the ADNEX model to our training and validation sets and computed the risk of malignancy using a web‐based calculator which was available on the IOTA website (www.iotagroup.org) at the time of data analysis. The sensitivity and specificity of the ADNEX model were calculated using two risk thresholds for malignancy, i.e. 10% and 20%. The 10% risk threshold is recommended in a European consensus statement^38^. The 20% risk cut‐off was chosen to improve specificity. We also calculated sensitivity at a specificity of 0.90, and specificity at a sensitivity of 0.90 for all models.

The statistical significance of any difference in AUC between the two radiomics nested models was tested using the F‐test method^39^, and that of differences in AUC between the radiomics models and the ADNEX model was calculated using the DeLong method^40^.

#### Analysis of reliability of radiomics features

To evaluate the reliability of radiomics feature values when analyzing different images from the same tumor, we calculated the intraclass correlation coefficients (ICC) based on a mean‐rating (k = 4), absolute‐agreement, two‐way mixed‐effects model, using SPSS statistical package version 23 (IBM Corp., Armonk, NY, USA). We included tumors of both the training and validation sets for which at least four images were available, and we analyzed four images per tumor. For tumors with more than four images available, four images from each tumor were selected randomly using the RANDBETWEEN function in an Excel worksheet containing information on all images. Koo and Li^41^ proposed that ICC values < 0.5 indicate poor reliability, values ≥ 0.5 and < 0.75 moderate reliability, values ≥ 0.75 and < 0.9 good reliability and values ≥ 0.9 excellent reliability.

#### Analysis of differences in radiomics features between right and left solid tumors with the same histology

In case of bilateral solid adnexal tumors with the same histology, the mean value of each radiomics feature was first computed from all available images (one or more) of the same tumor. Then, a paired *t*‐test was carried out to check if the values for any of the radiomics features differed statistically significantly between the right and left masses.

## RESULTS

From February 2014 to January 2021, there were 574 patients with one or more solid adnexal masses examined at Fondazione Policlinico Universitario A. Gemelli IRCCS, Rome, Italy. Of these, 242 were excluded, because digital ultrasound images were not available (*n* = 208) or digital images were not suitable for radiomics analysis, either because calipers or text covered the tumor or because the external margins of the ovarian tumor were not well‐visualized (*n* = 34). For the remaining 332 patients, DICOM images were available, but six of them were excluded because the files were compromised during the export phase in the contouring process, so that certain data became inaccessible for analysis. Thus, 326 patients were included in the final analysis (228 in the training set and 98 in the validation set). A total of 775 preoperative ultrasound images were used in the analysis. Overall, 276 (84.7%) patients (191 in the training set and 85 in the validation set) had only transvaginal images, 27 (8.3%) patients (18 in the training set and nine in the validation set) had only transabdominal images and 23 (7.1%) patients had both transvaginal and transabdominal images.

### Clinical, ultrasound and histopathological data

Clinical characteristics of the patients in the training and test sets are summarized in Table 1. The median age of the total study population was 56 (range, 18–87) years and most patients were postmenopausal (204/326, 62.6%). The ultrasound characteristics of the tumors in the training and test sets are shown in Table 2, histological outcome in Table 3 and FIGO stage of primary malignant tumors in Table 4. Tumor size (largest diameter of the tumor as measured with ultrasound) ranged from 13 to 290 mm. Ultrasound features differed between the benign and malignant solid tumors in both the training and validation sets, with ascites and bilaterality being more common and acoustic shadowing less common in malignant than in benign tumors, and color score being higher in malignant than in benign tumors. Of the 326 patients, 83 (25.4%) patients had a benign tumor, 197 (60.4%) patients had a primary malignant tumor, of which 10 (5.1%) were borderline tumors, and 46 (14.1%) patients had a metastatic tumor. The most common benign diagnosis was ovarian fibroma (45/83, 54.2%). The most common type of primary invasive tumor was high‐grade serous ovarian cancer (139/187, 74.3%). The most common primary tumors in ovarian metastases were gastric cancer (10/46, 21.7%), colon cancer (7/46, 15.2%) and breast cancer (6/46, 13.0%). The distribution of specific histology and FIGO stages was similar in the training and validation sets, but borderline tumors were more common in the validation set (4/228, 1.8% *vs* 6/98, 6.1%) (Table 3).

**Table 1:** Characteristics of patients with solid adnexal mass (training and validation sets)

						Paitents with malignant tumor					
	All patients			Patients with benign tumor		Primary malignant tumor*		Metastatic tumor	
Total	Training	Validation	Training	Validation	Training	Validation	Training	Validation	*P* †	
Characteristic	(*n* = 326)	(*n* = 228)	(*n* = 98)	(*n* = 58)	(*n* = 25)	(*n* = 134)	(*n* = 63)	(*n* = 36)	(*n* = 10)	Training	Validation
Age at diagnosis (years)	56 (47–65) [18–87]	55 (46–64) [18–87]	60 (50–68) [18–85]	53 (38–60) [20–81]	60 (50–68) [23–82]	55 (47–65) [18–87]	61 (51–67) [18–85]	56 (47–66) [29–74]	59 (54–69) [48–80]	0.085‡	0.954‡
Nulliparous	88 (27.0)	66 (28.9)	22 (22.4)	21 (36.2)	9 (36.0)	38 (28.4)	12 (19.0)	7 (19.4)	1 (10.0)	0.21§	0.14§
Postmenopausal	204 (62.6)	135 (59.2)	69 (70.4)	32 (55.2)	16 (64.0)	82 (61.2)	45 (71.4)	21 (58.3)	8 (80.0)	0.73§	0.62§
Symptomatic	206 (63.2)	147 (64.5)	59 (60.2)	23 (39.7)	7 (28.0)	101 (75.4)	45 (71.4)	23 (63.9)	7 (70.0)	< 0.0001§	< 0.0001§
CA125 (U/mL)	143.6 (20.6–657.3) [5.0–12 114.0]	138.9 (20.0–716.8) [5.0–12 114.0]	166.0 (26.8–497.5) [5.0–5982.0]	10.8 (9.8–21.9) [6.0–256.0]	10.6 (9.2–75.3) [5.0–508.2]	387.0 (94.2–1149.0) [5.0–12 114.0]	385.1 (90.0–830.0) [5.0–5982.0]	63.6 (18.0–210.7) [5.0–2200.0]	249.0 (102.4–336.0) [10.4–1168.0]	< 0.0001‡	< 0.0001‡

Categorical data are reported as *n* (%) and quantitative variables as median (interquartile range) [range].

* Including both borderline and primary invasive tumors.

† Comparison across three categories, i.e. benign *vs* primary malignant *vs* metastatic.

‡ Kruskall–Wallis test.

§ Chi‐square test.

**Table 2:** Ultrasound characteristics of tumors in training and validation sets, as recorded in International Ovarian Tumor Analysis database

						Malignant tumors					
	All tumors			Benign tumors		Primary malignant tumors*		Metastatic tumors	
Total	Training	Validation	Training	Validation	Training	Validation	Training	Validation	*P* †	
Characteristic	(*n* = 326)	(*n* = 228)	(*n* = 98)	(*n* = 58)	(*n* = 25)	(*n* = 134)	(*n* = 63)	(*n* = 36)	(*n* = 10)	Training	Validation
Ascites	97 (29.8)	71 (31.1)	26 (26.5)	0 (0)	1 (4.0)	56 (41.8)	22 (34.9)	15 (41.7)	3 (30.0)	< 0.0001‡	< 0.0001‡
Bilaterality	136 (41.7)	89 (39.0)	47 (48.0)	6 (10.3)	3 (12.0)	68 (50.7)	38 (60.3)	15 (41.7)	6 (60.0)	< 0.0001‡	< 0.0001‡
Largest diameter (mm)	66 (44–96) [13–290]	69 (46–99) [13–290]	60 (42–91) [19–242]	44 (29–62) [13–140]	48 (38–65) [19–194]	74 (51–106) [18–250]	62 (47–96) [21–242]	94 (76–126) [30–290]	62 (38–118) [30–229]	< 0.0001§	0.26§
Acoustic shadows	70 (21.5)	48 (21.1)	22 (22.4)	37 (63.8)	19 (76.0)	9 (6.7)	2 (3.2)	2 (5.6)	1 (10.0)	< 0.0001‡	< 0.0001‡
Papillary projection	10 (3.1)	7 (3.1)	3 (3.1)	2 (3.4)	0 (0)	5 (3.7)	3 (4.8)	0 (0)	0 (0)	0.51‡	0.42‡
Color score										< 0.0001‡	< 0.0001‡
1 (no blood flow)	19 (5.8)	12 (5.3)	7 (7.1)	9 (15.5)	6 (24.0)	2 (1.5)	0 (0)	1 (2.8)	1 (10.0)	—	—
2 (minimal blood flow)	69 (21.2)	47 (20.6)	22 (22.4)	32 (55.2)	12 (48.0)	13 (9.7)	7 (11.1)	2 (5.6)	3 (30.0)	—	—
3 (moderate blood flow)	136 (41.7)	99 (43.4)	37 (37.8)	13 (22.4)	5 (20.0)	69 (51.5)	30 (47.6)	17 (47.2)	2 (20.0)	—	—
4 (very strong blood flow)	102 (31.3)	70 (30.7)	32 (32.7)	4 (6.9)	2 (8.0)	50 (37.3)	26 (41.3)	16 (44.4)	4 (40.0)	—	—

Categorical data are reported as *n* (%) and quantitative variables as median (interquartile range) [range].

* Including both borderline and primary invasive tumors.

† Comparison across three categories, i.e. benign *vs* primary malignant *vs* metastatic.

‡ Chi‐square test.

§ Kruskall–Wallis test.

**Table 3:** Histological outcomes in training and validation sets

						Malignant tumors			
	All tumors			Benign tumors		Primary malignant tumors*		Metastatic tumors	
Characteristic	Total (*n* = 326)	Training (*n* = 228)	Validation (*n* = 98)	Training (*n* = 58)	Validation (*n* = 25)	Training (*n* = 134)	Validation (*n* = 63)	Training (*n* = 36)	Validation (*n* = 10)
Benign	83 (25.5)	58 (25.4)	25 (25.5)	58 (100)	25 (100)	NA	NA	NA	NA
Fibroma	45 (13.8)	29 (12.7)	16 (16.3)	29 (50.0)	16 (64.0)	NA	NA	NA	NA
Serous adenofibroma	11 (3.4)	8 (3.5)	3 (3.1)	8 (13.8)	3 (12.0)	NA	NA	NA	NA
Brenner tumor	7 (2.1)	5 (2.2)	2 (2.0)	5 (8.6)	2 (8.0)	NA	NA	NA	NA
Other benign	20 (6.1)	16 (7.0)	4 (4.1)	16 (27.6)	4 (16.0)	NA	NA	NA	NA
Borderline tumor	10 (3.1)	4 (1.8)	6 (6.1)	NA	NA	4 (3.0)	6 (9.5)	NA	NA
Primary invasive tumor	187 (57.4)	130 (57.0)	57 (58.2)	NA	NA	130 (97.0)	57 (90.5)	NA	NA
High‐grade serous	139 (42.6)	98 (43.0)	41 (41.8)	NA	NA	98 (73.1)	41 (65.1)	NA	NA
Endometrioid	14 (4.3)	10 (4.4)	4 (4.1)	NA	NA	10 (7.5)	4 (6.3)	NA	NA
Other malignant	34 (10.4)	22 (9.6)	12 (12.2)	NA	NA	22 (16.4)	12 (19.0)	NA	NA
Metastases from other tumors	46 (14.1)	36 (15.8)	10 (10.2)	NA	NA	NA	NA	36 (100)	10 (100)
Gastric cancer	10 (3.1)	8 (3.5)	2 (2.0)	NA	NA	NA	NA	8 (22.2)	2 (20.0)
Colon cancer	7 (2.1)	5 (2.2)	2 (2.0)	NA	NA	NA	NA	5 (13.9)	2 (20.0)
Breast cancer	6 (1.8)	3 (1.3)	3 (3.1)	NA	NA	NA	NA	3 (8.3)	3 (30.0)
Endometrial cancer	6 (1.8)	6 (2.6)	0 (0)	NA	NA	NA	NA	6 (16.7)	0 (0)
Other	17 (5.2)	14 (6.1)	3 (3.1)	NA	NA	NA	NA	14 (38.9)	3 (30.0)

Data are shown as *n* (%).

* Including both borderline and primary invasive tumors. NA, not applicable.

**Table 4:** FIGO stage of primary malignant tumors (*n* = 187)*

	Primary malignant tumors†		
FIGO stage	All (*n* = 187)	Training (*n* = 130)	Validation (*n* = 57)
I–II	53 (28.3)	33 (25.4)	20 (35.1)
III–IV	134 (71.7)	97 (74.6)	37 (64.9)

Data are shown as *n* (%).

* Data missing in 10 cases (four from training set and six from validation set).

† Including both borderline and primary invasive tumors. FIGO, International Federation of Gynecology and Obstetrics^28^.

Bilateral solid tumors with the same histology were found in 136/326 (41.7%) patients, including in 89/228 (39.0%) patients in the training set and in 47/98 (48.0%) in the validation set. In the training set, 6/58 (10.3%) benign tumors, 68/134 (50.7%) primary malignant tumors and 15/36 (41.7%) metastatic tumors were bilateral. In the validation set, 3/25 (12.0%) benign tumors, 38/63 (60.3%) primary malignant tumors and 6/10 (60.0%) metastatic tumors were bilateral.

### Radiomics feature analysis

We extracted a total of 68 radiomics features from each ROI. When comparing benign with malignant (including borderline, primary invasive and metastatic) tumors in the training set, 52 features were statistically significantly different (Table S2). The same 52 radiomics features differed statistically significantly between benign, borderline, primary invasive and metastatic tumors. However, the calculated values of each feature did not differ statistically significantly between primary malignant (including borderline and primary invasive) and metastatic tumors.

At least four images were available for 54/326 (16.6%) tumors (for 38 tumors these were only transvaginal images, for two tumors only transabdominal images and for 14 tumors both transvaginal and transabdominal images) and these were included in the analysis of intratumor reliability of radiomics features. Table S3 shows the results. The reliability across different images of the same lesion was moderate for eight features (ICC ranging from 0.516 to 0.743), good for 17 (ICC ranging from 0.750 to 0.899) and excellent for 34 (ICC ranging from 0.912 to 1). Kurtosis (F.stat_kurt) was the only feature that had poor reliability (ICC of 0.089). Therefore, we excluded it as a covariate from the analyses.

There were 136 patients with bilateral solid tumors that had the same histology. No statistically significant differences were found between the radiomics features of the right and left solid adnexal masses (Table S4).

**Figure 3:**
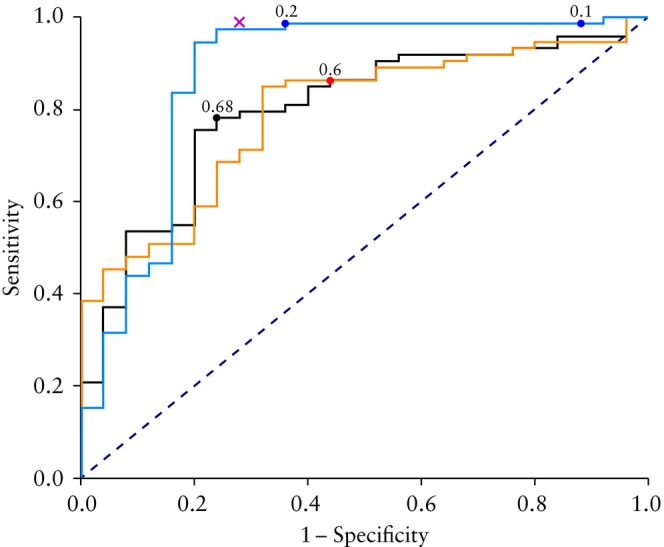
Receiver‐operating‐characteristics (ROC) curves summarizing performance in discriminating between benign and malignant solid adnexal masses of radiomics‐only model (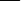), age + radiomics model (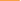) and Assessment of Different NEoplasias in the adneXa (ADNEX) model (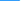) (including three clinical and six ultrasound features) in the validation set. Dashed line shows line of equality, i.e. if ROC curve classification was random. Circles show best cut‐offs, i.e. risk‐of‐malignancy cut‐offs yielding the highest proportion of correctly classified cases (according to Youden's index) for radiomics‐only model (0.68) and age + radiomics model (0.60), and for the 10% and 20% predefined malignancy risk cut‐offs for the ADNEX model. 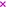 indicates classification performance of subjective assessment.

Among the 52 radiomics features that differed statistically significantly between benign and malignant tumors, 18 uncorrelated features (correlation coefficient > 0.9) were included in the models to predict malignancy (two statistical features (mean intensity, skewness of the intensity distribution); two textural features based on the GLCM (joint maximum, cluster shade); six textural features based on the GLRLM (gray‐level non‐uniformity, short runs emphasis, normalized run length non‐uniformity, long run high gray‐level emphasis, run entropy, long runs emphasis); and eight textural features based on the GLSZM (zone size non‐uniformity, gray‐level non‐uniformity, zone percentage, normalized zone size non‐uniformity, small zone emphasis, large zone high gray‐level emphasis, zone size variance, small zone high gray‐level emphasis). The short names of these 18 uncorrelated radiomics features are available in Appendix S1, ^30^. The discriminative performance of the two radiomics models and that of the ADNEX model is shown in Table 5 and Figure 3. In the training set, adding age to the radiomics‐only model improved discrimination between benign and malignant tumors (AUC of 0.86 for the radiomics‐only model *vs* 0.92 for the age + radiomics model). However, on internal validation, the AUC of the radiomics‐only model decreased from 0.86 to 0.80 and that of the radiomics + age model decreased from 0.92 to 0.79, so that the AUCs of the two radiomics models were similar in the validation set (*P* = 0.32). In the validation set, the AUC of the ADNEX model was greater than that of the radiomics‐only model (0.88 *vs* 0.80; *P* = 0.16) and that of the age + radiomics model (0.88 *vs* 0.79; *P* = 0.10).

**Table 5:** Comparison of performance in discriminating between benign and malignant solid adnexal masses of radiomics models; Assessment of Different NEoplasias in the adneXa (ADNEX) model and subjective assessment by an experienced ultrasound examiner, in training (228 patients) and validation (98 patients) sets

Model	AUC	Accuracy	Sensitivity	Specificity	LR+	LR−
Radiomics‐only (Youden's cut‐off: 68%)						
Training	0.86 (0.81–0.91)	0.78 (0.73–0.83)	0.78 (0.72–0.84)	0.79 (0.69–0.88)	3.75 (1.70–7.15)	0.28 (0.15–0.57)
Validation	0.80 (0.70–0.90)	0.78 (0.69–0.86)	0.78 (0.69–0.88)	0.76 (0.59–0.93)	3.25 (1.11–9.51)	0.29 (0.10–0.84)
Age + radiomics (Youden's cut‐off: 60%)						
Training	0.92 (0.89–0.96)	0.88 (0.85–0.93)	0.92 (0.90–0.97)	0.76 (0.63–0.85)	3.83 (1.55–8.44)	0.10 (0.04–0.20)
Validation	0.79 (0.69–0.89)	0.79 (0.70–0.87)	0.86 (0.78–0.94)	0.56 (0.36–0.75)	1.96 (0.70–5.51)	0.24 (0.09–0.69)
ADNEX (Cut‐off: 10%)						
Training	0.93 (0.89–0.97)	0.82 (0.77–0.87)	0.99 (0.98–1.00)	0.29 (0.18–0.41)	1.41 (0.18–10.87)	0.02 (0.00–0.16)
Validation	0.88 (0.78–0.97)	0.77 (0.68–0.85)	0.99 (0.96–1.00)	0.12 (0.00–0.25)	1.12 (0.11–11.33)	0.11 (0.01–1.15)
ADNEX (Cut‐off: 20%)						
Training	0.93 (0.89–0.97)	0.86 (0.81–0.90)	0.96 (0.93–0.99)	0.55 (0.42–0.68)	2.14 (0.86–5.35)	0.07 (0.03–0.19)
Validation	0.88 (0.78–0.97)	0.90 (0.84–0.96)	0.99 (0.96–1.00)	0.64 (0.45–0.83)	2.74 (0.32–23.19)	0.02 (0.00–0.18)
Subjective assessment						
Training	NA	0.92 (0.88–0.95)	0.97 (0.95–1.00)	0.76 (0.65–0.87)	4.02 (1.37–11.77)	0.04 (0.01–0.11)
Validation	NA	0.92 (0.86–0.97)	0.99 (0.96–1.00)	0.72 (0.54–0.90)	3.52 (0.41–30.48)	0.02 (0.00–0.16)

Values in parentheses are 95% CIs constructed using bootstrap for area under the receiver‐operating‐characteristics curve (AUC) and normal approximation for all other measures. LR+, positive likelihood ratio; LR−, negative likelihood ratio; NA, not applicable.

Classification performance (sensitivity and specificity at selected cut‐offs) of the models and of subjective assessment is presented in Table 5. In the validation set, subjective assessment had the best classification performance (sensitivity, 0.99 and specificity, 0.72), the ADNEX model at the 20% risk‐of‐malignancy cut‐off had the second best (sensitivity, 0.99 and specificity, 0.64) and the age + radiomics model had the poorest classification performance (sensitivity, 0.86; specificity, 0.56 at the 60% risk cut‐off). Tables S5 and S6 show classification performance at a fixed sensitivity of 0.90 and at a fixed specificity of 0.90 in the validation set. At a fixed sensitivity of 0.90, the ADNEX model had the highest specificity (0.80). At a fixed specificity of 0.90, the ADNEX model had the lowest sensitivity (0.44).

The radiomics models performed better when validated on tumors for which multiple images were available (*n* = 56) compared to when validated on tumors for which only one image was available (*n* = 42) (Table S7).

## DISCUSSION

We have shown that radiomics features differ between benign and malignant solid adnexal masses, and we built machine‐learning models including radiomics features that can be used to discriminate between benign and malignant solid adnexal masses. A model including 18 uncorrelated radiomics features had moderate discriminative ability on internal validation. The discriminative ability of the model including age and radiomics features was not superior to that of the radiomics‐only model.

To our knowledge, this is the largest study to date on radiomics analysis of ultrasound images of solid adnexal tumors. Important limitations are the retrospective, single‐center study design and the lack of external validation. Moreover, intra‐ and inter‐rater variability of the segmentation was not estimated. Another limitation is that we did not include morphological radiomics features (i.e. area, elongation, roughness), because the analysis was performed on two‐dimensional images. On the other hand, this is an advantage for the application of the model in a clinical setting, in which three‐dimensional ultrasound images are not always available. It is a limitation that calibration/goodness‐of‐fit tests were not performed.

In our study, 75% of the solid masses were malignant. This is a higher prevalence of malignancy than that reported in the studies of Timmerman *et al*.^1^ and Chiappa *et al*.^23^ (both 60%), both studies including masses examined in referral centers and removed by surgery. However, the proportion of solid tumors that were metastatic was lower in our study (14%) than in that of Valentin *et al*.^2^ (30%). Yet, the latter used the same study cohort as Timmerman *et al*.^1^.

Other research groups have developed ultrasound‐based radiomics machine‐learning models to discriminate between benign and malignant adnexal masses. In a retrospective study by Chiappa *et al*.^23^, 95 solid, 66 cystic and 80 ‘solid‐cystic’ tumors were examined. Their model, which included exclusively radiomics features, had good discriminative performance (AUC, 0.87) when applied to solid tumors, but there is a risk that their model was overfitted (269 radiomics features, 95 solid masses). Chiappa and coworkers also developed a decision support system to discriminate between benign and malignant adnexal masses which was based on menopausal status, acoustic shadowing, CA125 and radiomics features^42^. An ultrasound‐based radiomics model for discriminating between benign and malignant ovarian serous tumors was developed by Qi *et al*.^43^. Their results are not comparable to ours because their study included a single histological type and did not include exclusively solid masses.

We had hoped that our radiomics models would have discriminative ability similar to that of the ADNEX model, i.e. that the radiomics features would be able to replace the ultrasound variables in the ADNEX model. However, the discriminative ability of our radiomics models was inferior to that of the ADNEX model. The ADNEX model is currently the best performing prediction model for ovarian malignancy^5^, ^44^. In our study, the ADNEX model had a classification performance (at a 20% risk‐of‐malignancy cut‐off) that was almost as good as that of subjective assessment by an experienced ultrasound examiner.

We are disappointed that we did not find any radiomics features that could discriminate between primary malignant and metastatic solid tumors. This might be explained by the small number of metastases in our study population. The ADNEX model has been shown to have rather poor ability to discriminate between stage‐II–IV primary invasive ovarian malignancy and metastases when applied to all types of adnexal mass (AUC, 0.66)^5^, but there are no published results on its ability to discriminate between different types of solid ovarian tumor.

Safe use of the ADNEX model requires at least moderate ultrasound skills, knowledge of the IOTA terminology^25^ and IOTA certification (https://www.iotagroup.org/iota‐online‐course). The advantage of radiomics analysis of ultrasound images is that it is much less dependent on ultrasound skill, and so may be useful for less experienced examiners. In the future, machine learning with automatic segmentation could be embedded into ultrasound machines, enabling assessment of malignancy risk without requiring an expert ultrasound examiner^45^, ^46^, ^47^, ^48^.

It was surprising that addition of age to the radiomics features did not improve performance of the model. The drop in performance of the age + radiomics model between the training and validation sets indicates that the model might have been overfitted regarding age. Further work is needed to improve the robustness of radiomics ultrasound features. Our models performed better when tested on tumors with multiple images than when tested on tumors with only one image. This might be explained by improved characterization of the lesion and noise removal by averaging feature values across different images. Preprocessing images to remove speckle noise, and intensity normalization, could improve robustness, as textural features are sensitive to noise and ultrasound‐machine characteristics. Studies are needed to understand the best methodology to use in radiomics analysis of ultrasound images, for example, whether to use preprocessed images (denoising and intensity normalization) or native ultrasound images.

In conclusion, a random‐forest classifier was trained successfully and internally validated to stratify solid ovarian masses into benign and malignant by using radiomics information extracted from ultrasound images. Even though the discriminative and classification ability of our radiomics models was inferior to that of the ADNEX model and subjective evaluation by an experienced ultrasound examiner, we believe our results are sufficiently promising to justify continued development of radiomics analysis of ultrasound images of adnexal masses.

## Supporting information


**Table S1** Classifiers used for building radiomics models
**Table S2** Features that differed statistically significantly between benign and malignant tumors in the training set
**Table S3** Intraclass correlation coefficients (ICC) for extracted radiomics features
**Table S4** Comparison of radiomics features of right and left solid ovarian masses with the same histology (*n* = 136)
**Table S5** Specificity at 0.90 sensitivity for all models (validation set)
**Table S6** Sensitivity at 0.90 specificity for all models (validation set)
**Table S7** Performance of radiomics‐only and age + radiomics models in patients with single *vs* multiple images (validation set)
**Appendix S1** Selected radiomics features

## Data Availability

Data available on request from the authors.
