# Supplementary material for: Radiomics analysis of ultrasound images to discriminate between benign and malignant adnexal masses with solid morphology on ultrasound
Source: Ultrasound Obstet Gynecol. 2025 Feb 2;65(3):353–63. doi: 10.1002/uog.27680 (PMC11872347; doi:10.1002/uog.27680)
Supplement: Supplementary file 1 — Table S1 Classifiers used for building radiomics models Table S2 Features that differed statistically significantly between benign and malignant tumors in the training set Table S3 Intraclass correlation coefficients (ICC) for extracted radiomics features Table S4 Comparison of radiomics features of right and left solid ovarian masses with the same histology (n = 136) Table S5 Specificity at 0.90 sensitivity for all models (validation set) Table S6 Sensitivity at 0.90 specificity for all models (validation set) Table S7 Performance of radiomics‐only and age + radiomics models in patients with single vs multiple images (validation set) Appendix S1 Selected radiomics features [file UOG-65-353-s001.docx]

**Supporting information**

**Table S1** Classifiers used for building radiomics models

|  | Radiomics-only model | | | | Age+radiomics model | | | |
| --- | --- | --- | --- | --- | --- | --- | --- | --- |
|  | **Logistic regression** | **Support Vector Machine** | **XGBoost** | **Random Forest** | **Logistic regression** | **Support Vector Machine** | **XGBoost** | **Random Forest** |
| AUC | 0.78 | 0.71 | 0.76 | 0.80 | 0.76 | 0.71 | 0.75 | 0.79 |
| Accuracy | 0.72 | 0.69 | 0.74 | 0.78 | 0.71 | 0.65 | 0.71 | 0.79 |
| Sensitivity | 0.71 | 0.68 | 0.82 | 0.78 | 0.73 | 0.66 | 0.78 | 0.86 |
| Specificity | 0.76 | 0.72 | 0.52 | 0.76 | 0.68 | 0.64 | 0.52 | 0.56 |
| LR+ | 2.97 | 2.45 | 1.71 | 3.25 | 2.27 | 1.83 | 1.63 | 1.96 |
| LR- | 0.38 | 0.44 | 0.34 | 0.29 | 0.40 | 0.54 | 0.42 | 0.24 |

**Table S2** Features that differed statistically significantly between benign and malignant tumors in the training set (univariate analysis with Wilcoxon–Mann–Whitney test and P-values after Benjamini and Hochberg correction). Red: statistical features; green: textural features from the size zone matrix; yellow: textural features from the run length matrix; gray: textural features from the co-occurrence matrix. Text in bold denotes the 18 uncorrelated features from the correlation analysis. These 18 uncorrelated features were used in model building.

| **Variable** | **p-value**  **corrected** | **Variable** | **p-value**  **corrected** | **Variable** | **p-value**  **corrected** |
| --- | --- | --- | --- | --- | --- |
| **F_szm.zsnu** | **4.10-09** | F_cm.inv.diff | 9.57e-06 | F_cm.clust.tend | 2.93e-05 |
| **F_szm.glnu** | **6.07-09** | F_cm.dissimilarity | 9.57e-06 | F_cm.auto.corr | 3.51e-05 |
| **F_rlm.glnu** | **6.55e-07** | F_cm.contrast | 9.57e-06 | F_rlm.hgre | 4.11e-05 |
| **F_rlm.sre** | **7.37e-07** | F_cm.diff.entr | 9.57e-06 | **F_cm.clust.shade** | **4.40e-05** |
| F_szm.z.entr | 6.21e-06 | F_cm.inv.var | 9.57e-06 | F_rlm.glnu.norm | 4.50e-05 |
| **F_szm.z.perc** | **6.21e-06** | F_cm.diff.avg | 9.57e-06 | F_rlm.gl.var | 4.50e-05 |
| F_rlm.rlnu | 6.21e-06 | F_cm.diff.var | 9.57e-06 | **F_rlm.rlnu.norm** | **6.06e-05** |
| F_stat.energy | 7.01e-06 | F_rlm.r.perc | 1.02e-05 | F_cm.info.corr.2 | 6.95e-05 |
| F_rlm.srhge | 7.22e-06 | **F_stat.skew** | **1.12e-05** | **F_szm.zsnu.norm** | **1.31e-03** |
| F_stat.uniformity | 7.22e-06 | F_cm.sum.avg | 2.67e-05 | **F_rlm.lrhge** | **1.45e-03** |
| F_stat.entropy | 7.22e-06 | F_cm.sum.entr | 2.67e-05 | **F_szm.sze** | **1.45e-03** |
| **F_stat.mean** | **7.22e-06** | F_cm.energy | 2.67e-05 | **F_szm.lzhge** | **2.77e-03** |
| F_stat.mad | 7.22e-06 | F_cm.joint.entr | 2.67e-05 | **F_szm.zs.var** | **3.21e-03** |
| F_stat.var | 7.22e-06 | F_cm.joint.var | 2.67e-05 | **F_szm.szhge** | **1.73e-02** |
| F_stat.rms | 8.29e-06 | F_cm.joint.avg | 2.67e-05 | **F_rlm.rl.entr** | **3.26e-02** |
| F_cm.inv.diff.mom.norm | 9.57e-06 | **F_cm.joint.max** | **2.67e-05** | **F_rlm.lre** | **3.54e-02** |
| F_cm.inv.diff.mom | 9.57e-06 | F_cm.clust.prom | 2.67e-05 |  |  |
| F_cm.inv.diff.norm | 9.57e-06 | F_cm.sum.var | 2.93e-05 |  |  |

**Table S3** Intraclass correlation coefficients (ICC) for extracted radiomics features. Text in bold denotes the 18 uncorrelated features used as covariates in model development. The colors are explained in the bottom right side.

| **Statistical feature** | **ICC average** |  | **Textural feature from the gray level co-occurrence matrix** | **ICC average** |  | **Textural feature from the gray level run length matrix** | **ICC average** |  | **Textural feature from the gray level size zone matrix** | **ICC average** |
| --- | --- | --- | --- | --- | --- | --- | --- | --- | --- | --- |
| F_stat.energy | 0.933 |  | F_cm.joint.entr | 0.932 |  | **F_rlm.lre** | **0.995** |  | F_szm.lze | 1 |
| F_stat.rms | 0.929 |  | F_cm.sum.entr | 0.932 |  | F_rlm.rl.var | 0.959 |  | **F_szm.z.perc** | **0.924** |
| F_stat.entropy | 0.926 |  | F_cm.info.corr.2 | 0.931 |  | **F_rlm.rlnu.norm** | **0.948** |  | **F_szm.zsnu** | **0.87** |
| F_stat.var | 0.923 |  | F_cm.energy | 0.93 |  | **F_rlm.rl.entr** | **0.936** |  | **F_szm.glnu** | **0.842** |
| F_stat.mad | 0.923 |  | F_cm.joint.var | 0.929 |  | F_rlm.r.perc | 0.929 |  | F_szm.z.entr | 0.838 |
| F_stat.uniformity | 0.923 |  | F_cm.sum.var | 0.928 |  | **F_rlm.sre** | **0.917** |  | F_szm.glnu.norm | 0.775 |
| **F_stat.mean** | **0.922** |  | F_cm.clust.tend | 0.928 |  | **F_rlm.glnu** | **0.899** |  | F_szm.gl.var | 0.775 |
| **F_stat.skew** | **0.527** |  | F_cm.diff.entr | 0.925 |  | F_rlm.hgre | 0.871 |  | F_szm.hgze | 0.75 |
| F_stat.kurt | 0.089 |  | F_cm.diff.var | 0.924 |  | F_rlm.rlnu | 0.861 |  | **F_szm.zs.var** | **0.743** |
|  |  |  | F_cm.diff.avg | 0.923 |  | F_rlm.srhge | 0.835 |  | **F_szm.sze** | **0.727** |
|  |  |  | F_cm.contrast | 0.923 |  | F_rlm.glnu.norm | 0.822 |  | **F_szm.lzhge** | **0.696** |
|  |  |  | F_cm.dissimilarity | 0.923 |  | F_rlm.gl.var | 0.822 |  | **F_szm.szhge** | **0.67** |
|  |  |  | F_cm.inv.diff | 0.923 |  | **F_rlm.lrhge** | **0.597** |  | **F_szm.zsnu.norm** | **0.516** |
|  |  |  | F_cm.inv.diff.norm | 0.923 |  |  |  |  |  |  |
|  |  |  | F_cm.inv.diff.mom | 0.923 |  |  |  |  |  |  |
|  |  |  | F_cm.inv.diff.mom.norm | 0.923 |  |  |  |  |  |  |
|  |  |  | F_cm.inv.var | 0.923 |  |  |  |  | **EXPLANATION OF COLORS** |  |
|  |  |  | **F_cm.joint.max** | **0.922** |  |  |  |  | poor reliability | ICC < 0.5 |
|  |  |  | F_cm.clust.prom | 0.912 |  |  |  |  | moderate reliability | 0.5 <= ICC < 0.75 |
|  |  |  | F_cm.joint.avg | 0.864 |  |  |  |  | good reliability | 0.75 <= ICC < 0.9 |
|  |  |  | F_cm.sum.avg | 0.864 |  |  |  |  | excellent reliability | 0.9 <= ICC |
|  |  |  | F_cm.auto.corr | 0.853 |  |  |  |  |  |  |
|  |  |  | **F_cm.clust.shade** | **0.77** |  |  |  |  |  |  |
|  |  |  | F_cm.corr | 0.759 |  |  |  |  |  |  |
|  |  |  | F_cm.info.corr.1 | 0.739 |  |  |  |  |  |  |

**Table S4** Comparison of radiomics features of right and left solid ovarian masses with the same histology (n = 136)

| **Feature** | **Left**  **(mean ± SD)** | **Right**  **(mean ±SD)** | **Paired t-test*** |
| --- | --- | --- | --- |
| F_stat.mean | 0.0038±0.0074 | 0.0025±0.0038 | 0.19 |
| F_stat.var | 0.0038±0.0071 | 0.0025±0.0037 | 0.19 |
| F_stat.skew | 67.14±71.31 | 86.11±106.77 | 0.13 |
| F_stat.kurt | 9223.4±9223.4 | 9223.4±9223.4 | 0.11 |
| F_stat.mad | 0.0075±0.0143 | 0.0050±0.0074 | 0.19 |
| F_stat.energy | 7959.7±9223.4 | 5483.9±8400.1 | 0.24 |
| F_stat.rms | 0.0436±0.0422 | 0.03722±0.0327 | 0.27 |
| F_stat.entropy | 0.030±0.047 | 0.022±0.029 | 0.22 |
| F_stat.uniformity | 0.992±0.014 | 0.995±0.007 | 0.19 |
| F_cm.joint.max | 0.973±0.0389 | 0.981±0.0235 | 0.17 |
| F_cm.joint.avg | 0.0287±0.050 | 0.0165±0.023 | 0.10 |
| F_cm.joint.var | 0.018±0.023 | 0.014±0.016 | 0.23 |
| F_cm.joint.entr | 0.120±0.129 | 0.103±0.105 | 0.39 |
| F_cm.diff.avg | 0.006±0.007 | 0.005±0.006 | 0.87 |
| F_cm.diff.var | 0.005±0.006 | 0.005±0.006 | 0.89 |
| F_cm.diff.entr | 0.036±0.034 | 0.035±0.033 | 0.92 |
| F_cm.sum.avg | 0.057±0.010 | 0.033±0.045 | 0.10 |
| F_cm.sum.var | 0.066±0.088 | 0.049±0.060 | 0.21 |
| F_cm.sum.entr | 0.114±0.123 | 0.097±0.099 | 0.37 |
| F_cm.energy | 0.959±0.052 | 0.967±0.038 | 0.27 |
| F_cm.contrast | 0.006±0.007 | 0.005±0.006 | 0.87 |
| F_cm.dissimilarity | 0.006±0.007 | 0.005±0.006 | 0.87 |
| F_cm.inv.diff | 0.997±0.003 | 0.997±0.003 | 0.87 |
| F_cm.inv.diff.norm | 0.997±0.003 | 0.997±0.003 | 0.87cont |
| Table S4: continued | | | |
| **Feature** | **Left**  **(mean ± SD)** | **Right**  **(mean ±SD)** | **Paired t-test*** |
| F_cm.inv.diff.mom | 0.997±0.003 | 0.997±0.003 | 0.87 |
| F_cm.inv.diff.mom.norm | 0.997±0.003 | 0.997±0.003 | 0.87 |
| F_cm.inv.var | 0.006±0.007 | 0.005±0.006 | 0.87 |
| F_cm.corr | 0.728±0.134 | 0.710±0.113 | 0.34 |
| F_cm.auto.corr | 0.026±0.048 | 0.014±0.020 | 0.09 |
| F_cm.clust.tend | 0.066±0.088 | 0.049±0.060 | 0.21 |
| F_cm.clust.shade | 0.056±0.081 | 0.066±0.071 | 0.54 |
| F_cm.clust.prom | 0.134±0.135 | 0.123±0.130 | 0.62 |
| F_cm.info.corr.1 | -0.568±0.119 | -0.545±0.087 | 0.20 |
| F_cm.info.corr.2 | 0.136±0.084 | 0.126±0.075 | 0.42 |
| F_rlm.sre | 0.047±0.018 | 0.046±0.021 | 0.64 |
| F_rlm.lre | 265019.7±47467.2 | 264901±46240.3 | 0.96 |
| F_rlm.hgre | 0.110±0.059 | 0.102±0.061 | 0.28 |
| F_rlm.srhge | 0.030±0.013 | 0.029±0.016 | 0.89 |
| F_rlm.lrhge | 35.7±106.1 | 10.6±20.9 | 0.13 |
| F_rlm.glnu | 1518.9±395.6 | 1567.2±531.4 | 0.45 |
| F_rlm.glnu.norm | 0.859±0.058 | 0.868±0.064 | 0.27 |
| F_rlm.rlnu | 302.8±71.0 | 311.1±86.6 | 0.31 |
| F_rlm.rlnu.norm | 0.226±0.010 | 0.226±0.010 | 0.76 |
| F_rlm.r.perc | 0.009±0.007 | 0.009±0.006 | 0.84 |
| F_rlm.gl.var | 0.058±0.024 | 0.055±0.027 | 0.27 |
| F_rlm.rl.var | 25851.7±5015.1 | 25615.0±4681.0 | 0.58 |
| F_rlm.rl.entr | 5.10±0.21 | 5.07±0.19 | 0.42 |
| F_szm.sze | 0.053±0.021 | 0.047±0.027 | 0.23cont |
| Table S4: continued | | | |
| **Feature** | **Left**  **(mean ± SD)** | **Right**  **(mean ±SD)** | **Paired t-test*** |
| F_szm.lze | 635057689686.4±  160600213776.9 | 636312526547.9±  160680824616.1 | 0.87 |
| F_szm.hgze | 0.271±0.050 | 0.281±0.033 | 0.24 |
| F_szm.szhge | 0.041±0.021 | 0.039±0.025 | 0.67 |
| F_szm.lzhge | 9223.4±9233.4 | 9223.4±9223.4 | 0.16 |
| F_szm.glnu | 17.40±14.97 | 21.83±23.40 | 0.10 |
| F_szm.glnu.norm | 0.917±0.046 | 0.922±0.038 | 0.49 |
| F_szm.zsnu | 1.844±0.854 | 1.969±1.147 | 0.34 |
| F_szm.zsnu.norm | 0.698±0.021 | 0.703±0.030 | 0.19 |
| F_zsm.z.perc | 0.00024±0.00022 | 0.00025±0.00022 | 0.78 |
| F_szm.gl.var | 0.042±0.023 | 0.039±0.019 | 0.49 |
| F_szm.zs.var | 95824256.3±  101677826.5 | 107362155.9±  148148684.5 | 0.65 |
| F_szm.z.entr | 1.429±0.374 | 1.385±0.466 | 0.43 |

*No correction for multiple testing.

**Table S5** Specificity at 0.90 sensitivity for all models (validation set)

|  | **Radiomics-only model** | **Age+radiomics model** | **ADNEX model** |
| --- | --- | --- | --- |
|  | **n = 98** | **n = 98** | **n = 98** |
| **Cutoff** | 0.53 | 0.53 | 0.50 |
| **Sensitivity** | 0.90 | 0.90 | 0.87 |
| **Specificity** | 0.48 | 0.36 | 0.81 |

n=number of cases.

**Table S6** Sensitivity at 0.90 specificity for all models (validation set)

|  | **Radiomics-only model** | **Age+radiomics model** | **ADNEX model** |
| --- | --- | --- | --- |
|  | **n = 98** | **n = 98** | **n = 98** |
| **Cutoff** | 0.82 | 0.85 | 0.88 |
| **Sensitivity** | 0.53 | 0.48 | 0.44 |
| **Specificity** | 0.92 | 0.92 | 0.92 |

n=number of cases. Sens=sensitivity, Spec=specificity

**Table S7** Performance of radiomics-only and Age+radiomics models in patients with single *vs* multiple images (validation set)

|  | Radiomics-only model | | | Age+radiomics model | | |
| --- | --- | --- | --- | --- | --- | --- |
|  | All patients | Single image | Multiple images | All patients | Single image | Multiple images |
| n | **98** | **42** | **56** | **98** | **42** | **56** |
| AUC | 0.80 | 0.74 | 0.83 | 0.79 | 0.75 | 0.82 |
| Accuracy | 0.78 | 0.64 | 0.71 | 0.79 | 0.62 | 0.71 |
| Sensitivity | 0.78 | 0.73 | 0.83 | 0.86 | 0.81 | 0.89 |
| Specificity | 0.76 | 0.80 | 0.71 | 0.56 | 0.50 | 0.57 |

**Appendix S1**

The selected radiomics features are classified based on the following feature families: intensity-based statistical features (F_stat.), textural features based on the gray level co-occurrence matrix (F_cm), textural features based on the gray level run length matrix (F_rlm), textural features based on the gray level size zone matrix (F_szm).

The full names of the selected radiomics features are available in the Image Biomarker Standardization Initiative (IBSI)^29^ reference manual as follows:

- Statistical features

| F_stat.mean | Mean intensity |
| --- | --- |
| F_stat.skew | Skewness of the intensity distribution |

- Textural features based on the gray level co-occurrence matrix

| F_cm.joint.max | Joint maximum |
| --- | --- |
| F_cm.clust.shade | Cluster shade |

- Textural features based on the gray level run length matrix

| F_rlm.glnu | Gray level non-uniformity |
| --- | --- |
| F_rlm.sre | Short runs emphasis |
| F_rlm.rlnu.norm | Normalized run length non-uniformity |
| F_rlm.lrhge | Long run high gray level emphasis |
| F_rlm.rl.entr | Run entropy |
| F_rlm.lre | Long runs emphasis |

- Textural features based on the gray level size zone matrix

| F_szm.zsnu | Zone size non-uniformity |
| --- | --- |
| F_szm.glnu | Gray level non-uniformity |
| F_szm.z.perc | Zone percentage |
| F_szm.zsnu.norm | Normalized zone size non-uniformity |
| F_szm.sze | Small zone emphasis |
| F_szm.lzhge | Large zone high gray level emphasis |
| F_szm.zs.var | Zone size variance |
| F_szm.szhge | Small zone high gray level emphasis |
